# Metal doped layered MgB_2_ nanoparticles as novel electrocatalysts for water splitting

**DOI:** 10.1038/s41598-021-83066-7

**Published:** 2021-02-08

**Authors:** Ebrahim Sadeghi, Naeimeh Sadat Peighambardoust, Masoumeh Khatamian, Ugur Unal, Umut Aydemir

**Affiliations:** 1 Koç University Boron and Advanced Materials Application and Research Center (KUBAM), 34450 Sariyer, Istanbul Turkey; 2 Graduate School of Sciences and Engineering, Koç University, 34450 Sariyer, Istanbul Turkey; 3 Inorganic Chemistry Department, Faculty of Chemistry, University of Tabriz, 5166616471 Tabriz, Iran; 4 Koç University Surface Science and Technology Center (KUYTAM), 34450 Sariyer, Istanbul Turkey; 5 Department of Chemistry, Koç University, 34450 Sariyer, Istanbul Turkey

**Keywords:** Electrocatalysis, Catalysis, Energy, Chemistry

## Abstract

Growing environmental problems along with the galloping rate of population growth have raised an unprecedented challenge to look for an ever-lasting alternative source of energy for fossil fuels. The eternal quest for sustainable energy production strategies has culminated in the electrocatalytic water splitting process integrated with renewable energy resources. The successful accomplishment of this process is thoroughly subject to competent, earth-abundant, and low-cost electrocatalysts to drive the oxygen evolution reaction (OER) and hydrogen evolution reaction (HER), preferably, in the same electrolyte. The present contribution has been dedicated to studying the synthesis, characterization, and electrochemical properties of newfangled electrocatalysts with the formal composition of Mg_1−*x*_*TM*_*x*_B_2_ (*x* = 0.025, 0.05, and 0.1; *TM* (transition metal) = Fe and Co) primarily in HER as well as OER under 1 M KOH medium. The electrochemical tests revealed that among all the metal-doped MgB_2_ catalysts, Mg_0.95_Co_0.05_B_2_ has the best HER performance showing an overpotential of 470 mV at − 10 mA cm^−2^ and a Tafel slope of 80 mV dec^−1^ on account of its high purity and fast electron transport. Further investigation shed some light on the fact that Fe concentration and overpotential for HER have adverse relation meaning that the highest amount of Fe doping (*x* = 0.1) displayed the lowest overpotential. This contribution introduces not only highly competent electrocatalysts composed of low-cost precursors for the water-splitting process but also a facile scalable method for the assembly of highly porous electrodes paving the way for further stunning developments in the field.

## Introduction

Disruption of the ecological balance by fossil fuels, their volatile prices, and the ever-growing energy demands have provided tremendous impetus to look for new, environmentally benign, abundant, and zero-emitting energy sources^1–4^. Recent studies show that it will be unavoidable to launch and accelerate energy development from conventional energy systems to novel and viable alternatives^5^. Hydrogen, a potential energy carrier, has been addressed as the future energy owing to its higher energy density (∼120 MJ/kg) compared to petroleum-based energy sources (∼45 MJ/kg)^4,6,7^, low weight, high abundance, and without leaving any carbon footprints of the combustion products^8–10^.

The current massive-scale production of hydrogen through steam reforming of natural gas and/or methanol consumes an enormous amount of energy and produces CO_2_, making this process less viable from a sustainability perspective^11–13^. Unconventional and environmentally-benign H_2_ generation approaches encompass photo/electrolysis of water supported by renewable energy sources (e.g., solar, geothermal)^3,14,15^. In fact, in large-scale hydrogen production, the advantages of electrochemical water splitting outweigh those of other conventional systems, including coal gasification and steam reforming of methane, because it has an enormous amount of sources and easy to use without CO_2_ emission^13,16^.

Since the invention of the voltaic pile, scientists have made plenty of attempts to ameliorate the approach of energy production from water^17^, but mass-scale implementation based on photo/electrochemical water splitting processes is the major bottleneck mainly because of the inherently sluggish kinetics of OER and HER^14,18,19^. Electrolysis of water is simply composed of two reactions, the so-called, hydrogen evolution reaction (HER) and oxygen evolution reaction (OER). The reduction of H^+^ ions occurs at the cathode (2H^+^(aq) + 2e^−^  → H_2_ (g)) and the oxidation of water takes place at the anode (2H_2_O(l) → O_2_(g) + 4H^+^(aq) + 4e^−^)^5,20^. The thermodynamic voltage of water splitting is 1.23 V at 25 °C and 1 atm which corresponds to an energy input of ΔG° = 237.1 kJ mol^−1^^12,21^, but much higher potentials—referred to as overpotential—are required to initiate practical water splitting^22,23^.

To minimize the overpotentials at the cathode and improve energy conversion efficiency, effective electrocatalysts must be employed^3,24^. To date, Pt-based materials have been proven the most promising electrocatalysts for HER^5,25,26^. Nevertheless, the shortage of sources on earth and high cost limit their widespread industrial applications^20,26,27^. Thus, developing competent, stable, and low-cost catalytic materials for HER is crucial, but so far remains a great challenge^28,29^.

In the last few years, plenty of non-precious transition metal compounds, namely sulfides (NiS_2_, FeS_2_, CoS_2_), phosphides (Ni_2_P, FeP, CoP), and selenides (CoSe_2_, FeSe_2_) have been found with excellent HER activities^10,15,16^. Recently, transition metal (Ni, Co, Fe, etc.)-based borides (TMBs) have sparked considerable attention in the fields of energy conversion and storage. An growing number of studies evince that some traits introduce TMBs as exceptional electrocatalysts for water splitting^29–31^. As compared to other TM-based components, TMBs possess some special attributes such as high chemical, thermal, and mechanical stability, significant catalytic activity, and conductivity for water splitting^5,6,8,19,32,33^. In this sense, the results of several recent studies confirm the potential of transition metal borides as dual-functional catalysts for water splitting^34^; yet, among TMBs, metal diborides (MDbs) have been poorly explored for this application.

Metal diborides known by the general formula of *M*B_2_, feature some intrinsic singular characteristics including hardness, chemical durability, high thermal conductivity, and high electrical conductivity^35,36^. So far, MgB_2_, as a representative of the large family of MDbs has been a well-known material for its superconductivity at low temperatures^37–40^. MgB_2_ has other attractive characteristics as regards practical use, such as the ability of doping effect, low cost, and abundance of its raw materials, all of which makes it an auspicious candidate for doping and subsequently utilizing in water splitting^38^. Moreover, a singular property of MgB_2_ is the evacuation of B 2p–s bands on the basis of a unique Mg–B interaction. Accordingly, for any MDb, the correlation between boron and the metal atoms plays a decisive role on effectiveness of its properties leading to a firm premise for effective application in water electrolysis^41^.

Herein we examine and report a very successful approach to substitute the divalent transition metals in Mg_1−*x*_*TM*_*x*_B_2_ (*x* = 0.025, 0.05, and 0.1; *TM* = Fe and Co) for the Mg^2+^ as primarily enhanced HER electrocatalysts under alkaline medium. It should be pointed out that the aforementioned electrocatalysts have also been tested as OER catalysts and the data are provided in the supporting information.

## Results and discussion

**Figure 1:**
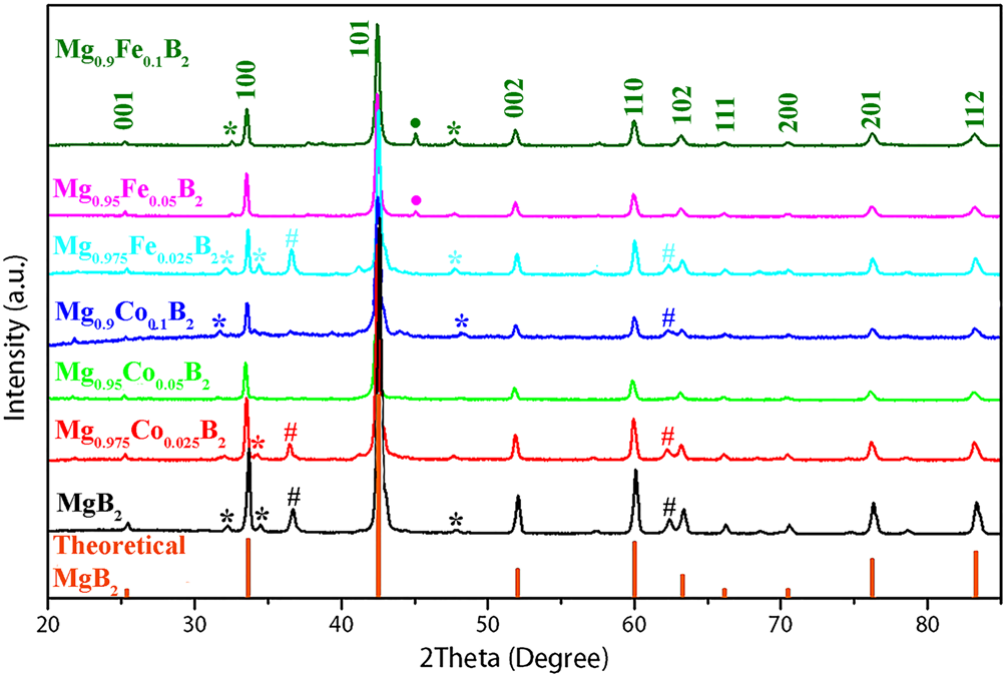
XRD patterns of MgB_2_ and Mg_1−*x*_*TM*_*x*_B_2_ (*x* = 0.025, 0.05, and 0.1; *TM* = Fe and Co): impurity peaks of Mg, MgO, and FeB/Fe_2_B are marked by *, #, and •, respectively.

XRD patterns of pure MgB_2_, Co-doped MgB_2_, and Fe-doped MgB_2_ are illustrated in Fig. 1. For comparison, the theoretical diffraction pattern of magnesium diboride has also been included^40,42^. Reflections shown in the theoretical pattern have appeared in both undoped MgB_2_ as well as Mg_1−*x*_*TM*_*x*_B_2_ which confirms the successful synthesis technique applied in this study. However, there are trace amounts of labeled intrinsic impurities such as MgO (marked by #) at 2θ of 36.7°, 62.38° and Mg (marked by *) at 32.28°, 34.52°, 47.86°. Based on the literature^40^, two main paths that lead to the formation of MgO are as follows.

- If Mg precursor powder is sintered under oxygen it will produce MgO according to the following reaction:
  $${\text{2Mg}}\left( {\text{s}} \right) + {\text{O}}_{{2}} \left( {\text{g}} \right) \to {\text{2MgO}}\left( {\text{s}} \right)$$
- Also, the reaction of B_2_O_3_ with Mg forms MgO based on the following reaction:
  $${\text{3Mg}}\left( {\text{s}} \right) + {\text{B}}_{{2}} {\text{O}}_{{3}} \to {\text{3MgO}}\left( {\text{s}} \right) + {\text{2B}}\left( {\text{s}} \right)$$

Because all the synthesis procedure was carried out in a glovebox, the probable occurrence of above-mentioned reactions can be attributed to the impurity of precursor materials. Therefore, it is ironically interesting that the raw materials of Mg powder have surface oxide to some extent that produces MgO which is utterly inevitable to prevent from happening.

The X-ray diffractograms of Co-doped MgB_2_ showed that there was no sign of secondary phases containing Co that is an obvious indication of successful substitution of Co for Mg in the crystal structure (see Figure S1 and Table S1). Nevertheless, weak peaks corresponding to Mg and MgO are still present with much lower intensity compared to MgB_2_.

The X-ray diffraction for the samples with formal composition Mg_1−*x*_Fe_*x*_B_2_ revealed that Mg and MgO peaks were roughly negligible. However, there were secondary phases at 2θ of 32.54°, 37.7°, and 45.08° which were assigned to the FeB phase. It is remarkable to mention that the impurity peak at 45.08° (labeled by •) can also be related to Fe_2_B, especially at high concentration doping of Fe (*x* = 0.1)^43^.

The minor amount of impurities with infinitesimal peaks indicated that the substitution of Co and Fe for Mg has significantly suppressed the amount of Mg and MgO formation. Moreover, with increasing Co and Fe content from *x* = 0.025 to 0.1, the amount of impurities decreases as well, leading to finer structures that are obviously as a result of lowering the amount of Mg in the composition. Altogether, Mg_0.95_Co_0.05_B_2_ contains the least amount of impurities.

The full-width-at-half-maximum (FWHM) values for synthesized catalysts were calculated by Program STOE WinXPOW (Darmstadt: Stoe & Cie GmbH, 2016) and subsequently crystallite size computed and summarized in Table 1. It is noteworthy to state that the FWHM of the XRD (101) peak has a direct relation with the concentration of Fe and Co dopants. This demonstrates that nano Fe- and Co-doped samples contain tiny grains and imperfect crystallinity. The Scherrer’s equation is applied to calculate the average particle size (D) of the nanoparticles for each sample (peak 101)

$${\text{D}} = \left( {k \lambda } \right){/}\left( {\beta \;\cos \theta } \right)$$

where k = 0.94 is the shape factor of a particle, presuming shape of the nanoparticles as sphere, λ = 1.5406 Å the wavelength of Cu Kα radiation, β is the FWHM of the XRD (101) peak, and θ is the diffraction angle of the peak^43^. A glance at the table points out that the average grain size of the parent MgB_2_ is about 29 nm. These results based on the calculation of the grain size of doped samples unveils that doping concentration and grain size have reverse relation. By increasing doping amounts in both Fe and Co compositions, grain size shrinks.

**Table 1:** FWHM values and crystallite size for pure Mg_1−*x*_*TM*_*x*_B_2_ (*x* = 0.025, 0.05, and 0.1; *TM* = Fe and Co).

Sample	FWHM (101)	D (nm)
MgB_2_	0.3087	28.82945
Mg_0.975_Fe_0.025_B_2_	0.2233	39.85514
Mg_0.95_Fe_0.05_B_2_	0.2447	36.36965
Mg_0.9_Fe_0.1_B_2_	0.2909	30.59351
Mg_0.975_Co_0.025_B_2_	0.2232	39.87299
Mg_0.95_Co_0.05_B_2_	0.2298	38.72782
Mg_0.9_Co_0.1_B_2_	0.3062	29.06483

**Figure 2:**
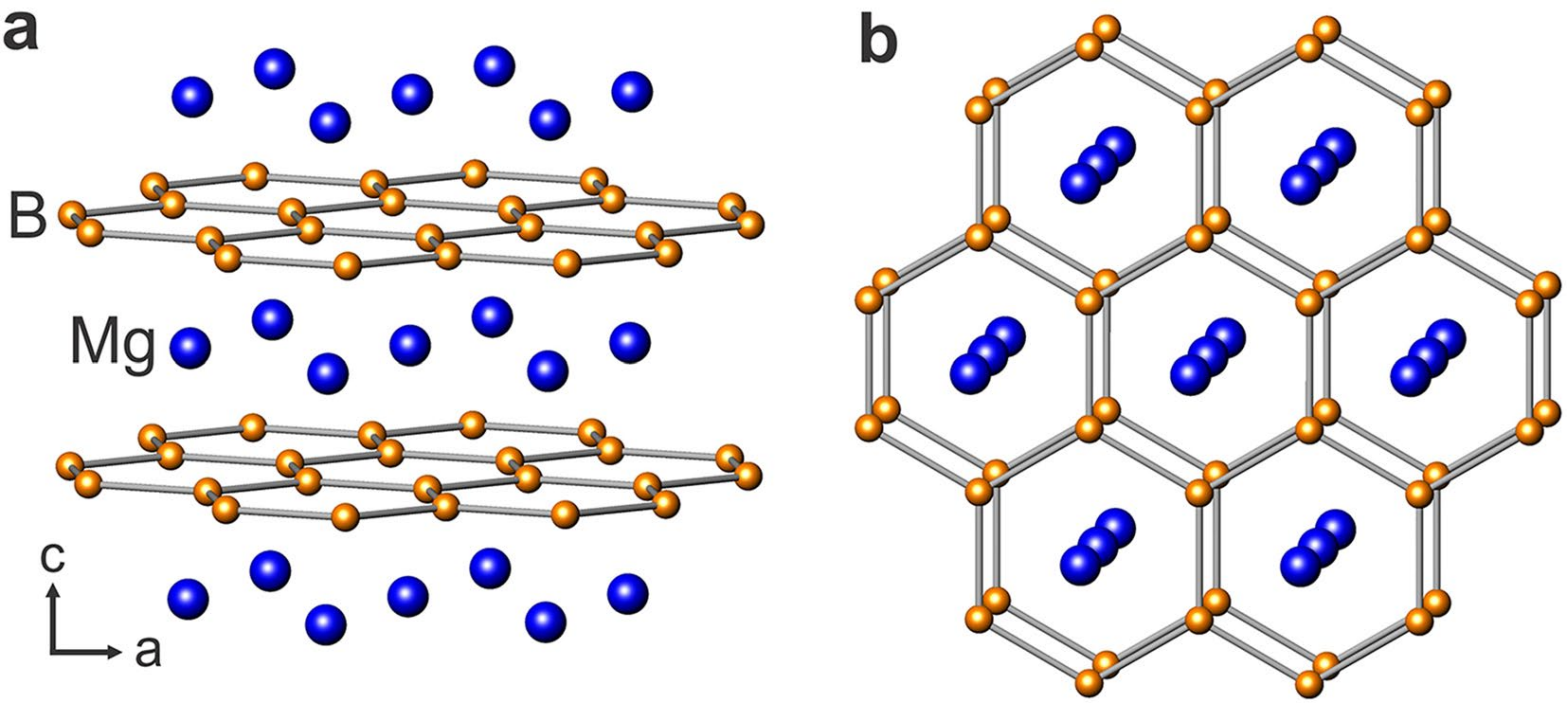
Schematic design of layered MgB_2_ structure. (**a**) Along the a-axis, (**b**) along the c-axis.

Regarding previous experimental and theoretical^44^ results, MgB_2_ crystallizes in the well-known AlB_2_-type structure with *P*6/*mmm* symmetry. As can be seen from Fig. 2, Mg layers are positioned between two honeycomb layers of B atoms. This structure resembles graphite regarded as that of completely intercalated graphite with carbon substituted by boron. Notwithstanding similarities, the B–B bonding is strongly anisotropic for the reason that intralayer B–B bonds are much shorter than the interlayer distance—which is not observed in graphite^44^.

Partial filling of two σ bands associated with strongly covalent sp^2^-hybrid bonding within the 2D boron layers is another attractive feature of MgB_2_. The crystal structure manifests that the holes at the top of these σ bands possess mainly two-dimensional properties and are restricted within the boron sheets. On the contrary, electrons and holes in the π bands mostly represent three-dimensional characteristic which are distributed through the whole crystal. Because of the extensive overlapping that takes place between all p orbitals (in-plane as well as out-of-plane) for adjacent boron atoms, σ and π bands encompass strong in-plane dispersion. However, the interlayer overlap, particularly for p_xy_ orbitals, are not large enough in such a way that the k_z_ dispersion of σ bands does not surpass 1 eV. It should be noted that unfilled σ bands in conjunction with boron p_xy_ character sufficiently maintain the covalent structure throughout the whole crystal^45^. Conducting covalent bonds stand for a distinctive feature of MgB_2_ turning it into an exceptional material probably existing at the threshold of structural instability^44^.

The refined lattice parameters of MgB_2_ and doped samples are tabulated in Table S2. The substitution of Mg^2+^ with Fe^2+^ and Co^2+^ leads to slightly higher lattice parameters due to the larger ionic radii of the transition metal ions (74.5 pm and 78 pm, respectively) compared to Mg^2+^ (72 pm)^46^.

**Figure 3:**
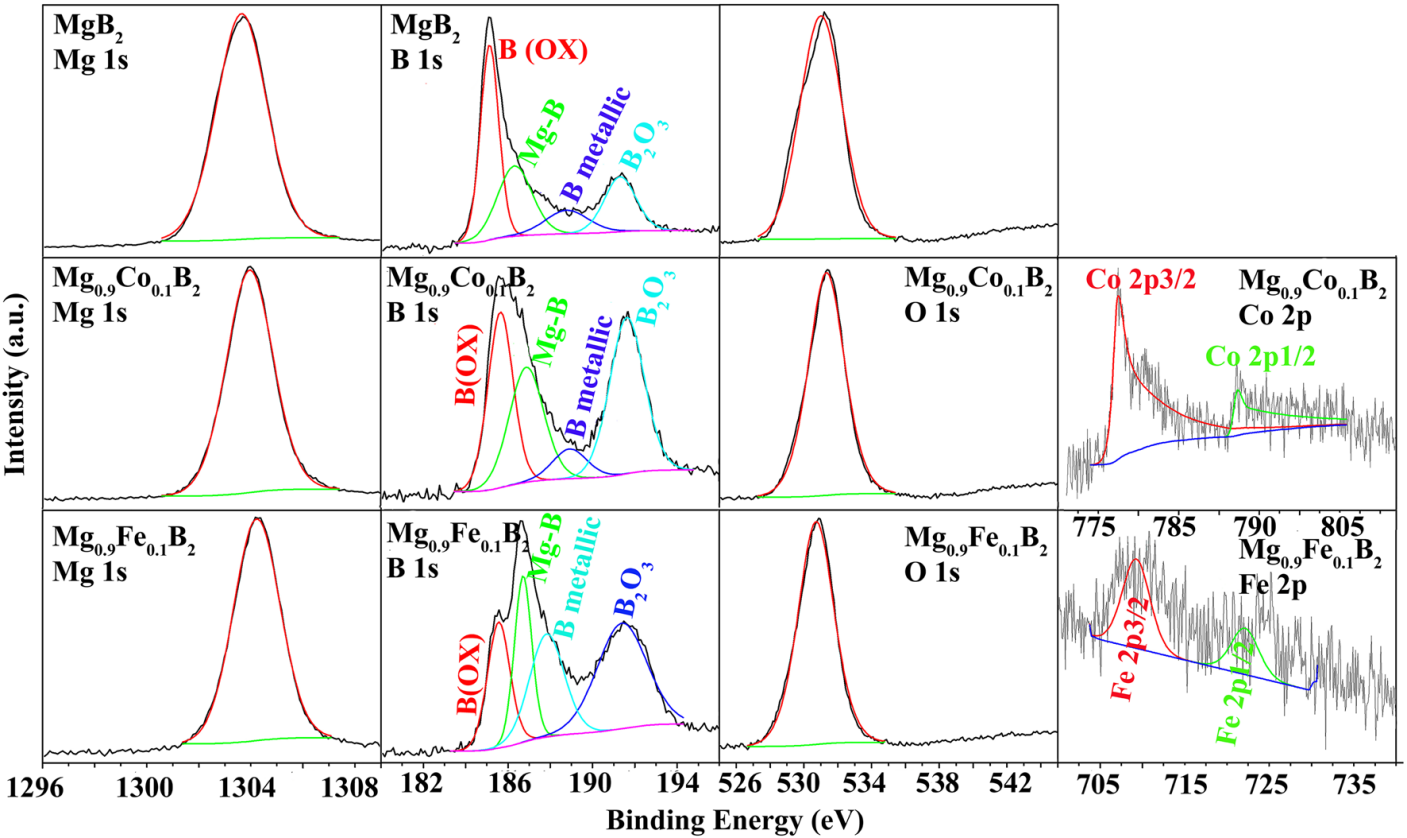
XPS spectra of Mg 1s, B 1s, O 1s, Co 2p, and Fe 2p in MgB_2_, Mg_0.9_Co_0.1_B_2_, and Mg_0.9_Fe_0.1_B_2_.

Figure 3 and S2 show XPS studies of pure MgB_2_, Co-doped MgB_2_, as well as Fe-doped MgB_2_ nanoparticles. For this, Mg 1s, B 1s, Co 2p, and Fe 2p spectra have been analyzed. Besides, O 1s spectra have also been considered explaining the trace amount of surface impurities such as MgO. Regarding the fact that the Ar atmosphere was applied in all synthesizing steps, the reaction of B_2_O_3_ with Mg (as mentioned in the previous section) is inevitable. So, MgB_2_ is somewhat vulnerable to oxidation as observed in Fig. 3 and S2 O 1s spectra. As it was stated in the literature, several authors, including Corneille et al.^47^ have reported two major peaks pertaining to O 1s and peroxide at 530.5 and 532–533.5 eV, separately. Likewise, several publications witnessed two-constituent peaks in the O 1s core-level spectrum, the lower binding energy peak at 531.0 ± 0.2 eV was ascribed to the MgO oxygen atom (O^2−^)^48^. The higher binding energy O 1s constituent at 533.2 eV was indexed to chemisorbed OH^−^ at MgO (100)^48^. Sanz et al.^49^ and Sanz et al.^50^ have also individually detected only one O 1s respective peaks at 531.2 and 530.5 eV, without any shoulder peak^51^. The emergence of any shoulder peak reported by some authors was on the account of their samples' growth procedures or conditions^51^. Considering the above-mentioned references, the major peak observed in this work corresponds to MgO at the binding energy of 531 eV. It should be noted that there is a minor shift in this peak position for Mg_0.9_Fe_0.1_B_2_, which falls within the accuracy of XPS measurements and data processing.

Fitting and deconvolution of Mg 1s spectra of pure MgB_2_, reveals a peak at 1303.7 eV. Based on the literature^52^, metallic Mg shows a peak of around 1303 eV. This positive shift of Mg 1s toward higher binding energies in MgB_2_ is related to donating electrons to boron; thereby, leading to stronger bonding in MgB_2_ compared to metallic Mg. Besides, in MgB_2_, there is a probability of some reverse charge transfer from B to Mg (+ 2) leading to a diminution of the formal charge of Mg and hence the partial covalency in the Mg–B bond. This charge transfer results in the fragmentary covalency in the Mg–B bond. As written above, in MgB_2_ structure—an example of a boron-rich system—the electrons transfer from Mg to B layers^29^. The electrons move to B π-bands as the σ-bands are filled, Then, some electrons shift from B π to Mg (+ 2). Consequently, the charge state of Mg is reduced from (+ 2) to lower states^45^.

A general trend can be observed in Fig. 3 and S2 that doping shifts Mg 1s toward higher binding energies. Specifically, in the low doping amount of Co and Fe (*x* = 0.025), Mg 1s peak shifts to even higher peaks, 1305.5 eV (refer to Figure S2). There are differences of > 1 eV in binding energies between doped and undoped samples. These large differences are ascribed to the effect of neighboring atoms. As stated by^52^, non-exchangeable and exchangeable Mg ions exhibit peaks around 1303 and 1306 eV, respectively. In low amounts of Co and Fe dopant (*x* = 0.025), Mg ions act as exchangeable ions, whereas, in higher contents of Co and Fe (*x* = 0.05 and 0.1), Mg ions are considered as non-exchangeable ones. For this case, the flow of electronic charge to Mg ion, and the extra-atomic relaxation from surrounding oxygen atoms are larger.

Figure 3 and S2 depict the examined B 1s spectra. B 1s spectra of pure MgB_2_ display peaks around 185.2, 186.4, 188.8, and 191.3 pertaining to B(O*X*), MgB_2_ (boron as boride anion), B as metallic, and B_2_O_3_, respectively. These are consistent with the binding energy values reported for Mg diboride^45^. MgB_2_ is viewed as Mg^2+^(B_2_)^2−^ with an average charge of (− 1) at B. As it was mentioned previously, in the MgB_2_ structure, the Mg charge is less than (+ 2), so the average charge on B is less than (− 1). Doping of MgB_2_ with metallic elements such as Co and Fe shifts binding energies of B to higher values, especially in the low amount of dopant as *x* = 0.025. This slight increase of binding energy stipulates the partial electron transfer from B to the vacant d-orbital of the metallic dopant.

To investigate the oxygen-containing compounds at the surface, the O 1s spectra have also been illustrated in Fig. 3 and S2. The prominent peak around 531 eV corresponds to MgO and surface oxygen possibility. Finally, Co 2p and Fe 2p peaks were observed in all doped samples, confirming the presence of Co and Fe species in the MgB_2_ structure. For Fe-doped Mg_1−*x*_*TM*_*x*_B_2_ (*x* = 0.025), Fe 2p_3/2_ peak is also possible, but it could not be deconvoluted due to its low intensity.

**Figure 4:**
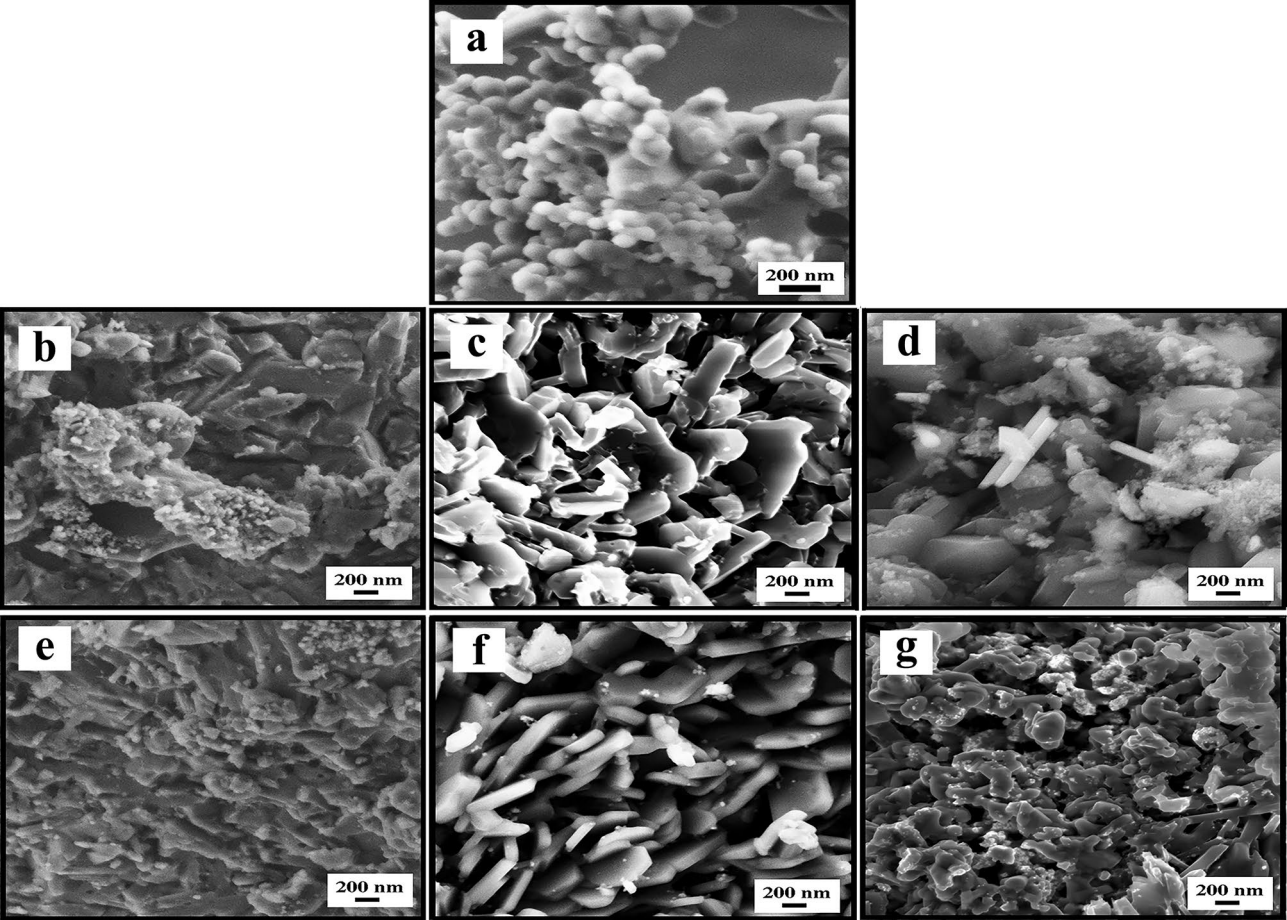
SEM images of (**a**) pure MgB_2_, (**b**) Mg_0.975_Co_0.025_B_2_, (**c**) Mg_0.95_Co_0.05_B_2_, (**d**) Mg_0.9_Co_0.1_B_2_, (**e**) Mg_0.975_Fe_0.025_B_2_, (**f**) Mg_0.95_Fe_0.05_B_2_, and (**g**) Mg_0.9_Fe_0.1_B_2_.

The EDS elemental mapping analyses performed at high-magnifications on Mg_0.95_Co_0.05_B_2_ and Mg_0.9_Fe_0.1_B_2_ indicate the homogenous distribution of the metal atoms throughout the grains of the target phases (Figure S3). Generally, the particle morphology studied by FESEM images (Fig. 4 and S4) disclosed that for pure MgB_2_, nanoparticle sizes are in the range of 40–300 nm, Fe-doped MgB_2_ 29–500 nm, and Co-doped MgB_2_ 39–450 nm. Needless to mention that larger particles with sizes as big as 1–2 µm were also detected which can be primarily because of agglomeration. The morphology of resulting particles demonstrated that pure MgB_2_ has been mostly dominated by spherical shape particles. Nonetheless, samples with a formal composition of Mg_1−*x*_*TM*_*x*_B_2_ displayed quite distinctive particle shapes (e.g., needle-like) that can be ascribed to interaction between metallic and boron layers as well as high pressure applied during their synthesis procedure. Comparing the individual crystallite of Fe and Co substituted MgB_2_ signifies the fact that Co-doped samples have slightly better incorporation of Co into the MgB_2_ structure. On the contrary, Fe-doped samples lack this benefit most probably due to the formation of secondary phases such as FeB or Fe_2_B. The mentioned conclusions were also witnessed in ref.^53^.

**Figure 5:**
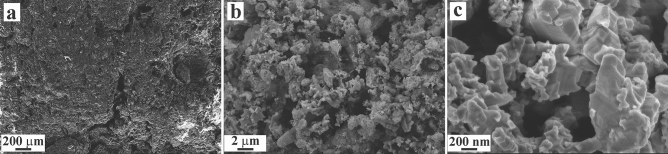
SEM images of the working electrode surface.

According to the previous literature^8,10,21,54^, catalysts with attributes such as large specific surface area, open porous structure, and high accessibility of active sites are expected to exhibit outstanding electrocatalytic performance. Thanks to the FESEM image of the surface of the working electrode (Fig. 5), uneven morphology, pores, and cracks can be observed that facilitates the accessibility of the electrolyte molecules into the far deeper parts of the electrode: thereby, drawing on remarkable catalytic activity.

**Figure 6:**
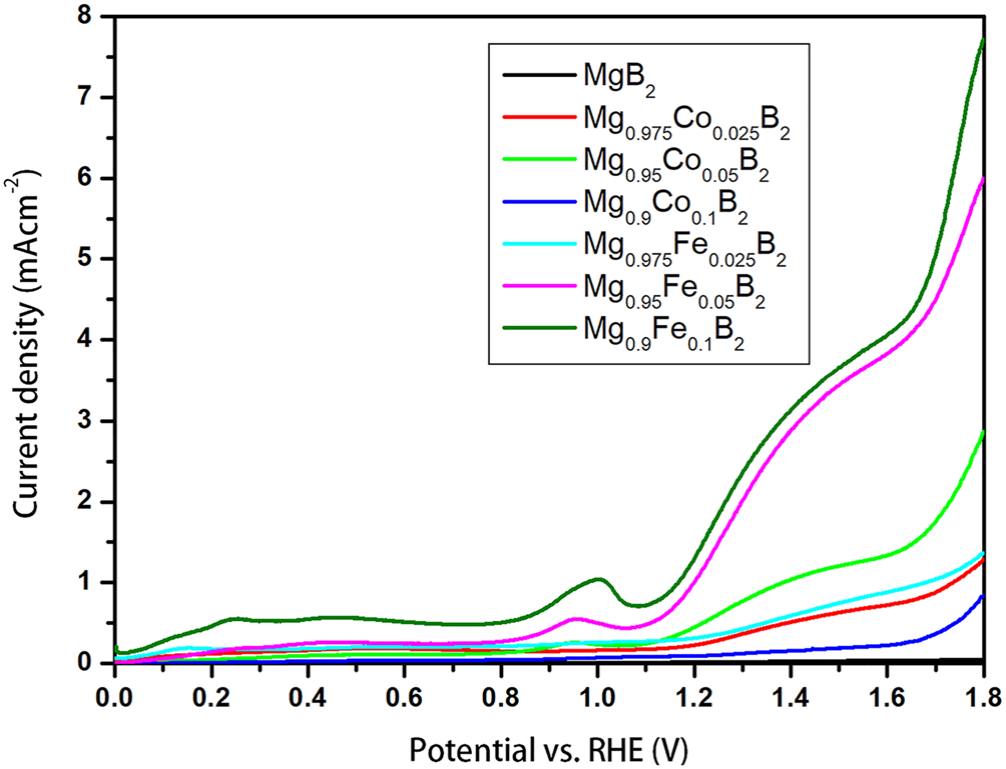
Polarization curves of the OER reaction of pure and doped MgB_2_.

Electrocatalytic features of undoped MgB_2_ and transition metal-doped MgB_2_ were evaluated using electrochemical tests. Both HER and OER were examined carefully. The recorded voltammograms of the OER are given in Fig. 6. As it is noticeable from this figure, Mg_0.9_Fe_0.1_B_2_ exhibits the superior reactivity amongst all the doped MgB_2_ samples. This decent performance should be assigned to the incorporation of Fe atoms into the MgB_2_ structure. In addition, as reported by Li et al.^55^ on boride-based electrodes, Fe dopants can enhance the local electronic structure owing to its superconductivity, resulting in enhanced OER performance.

**Figure 7:**
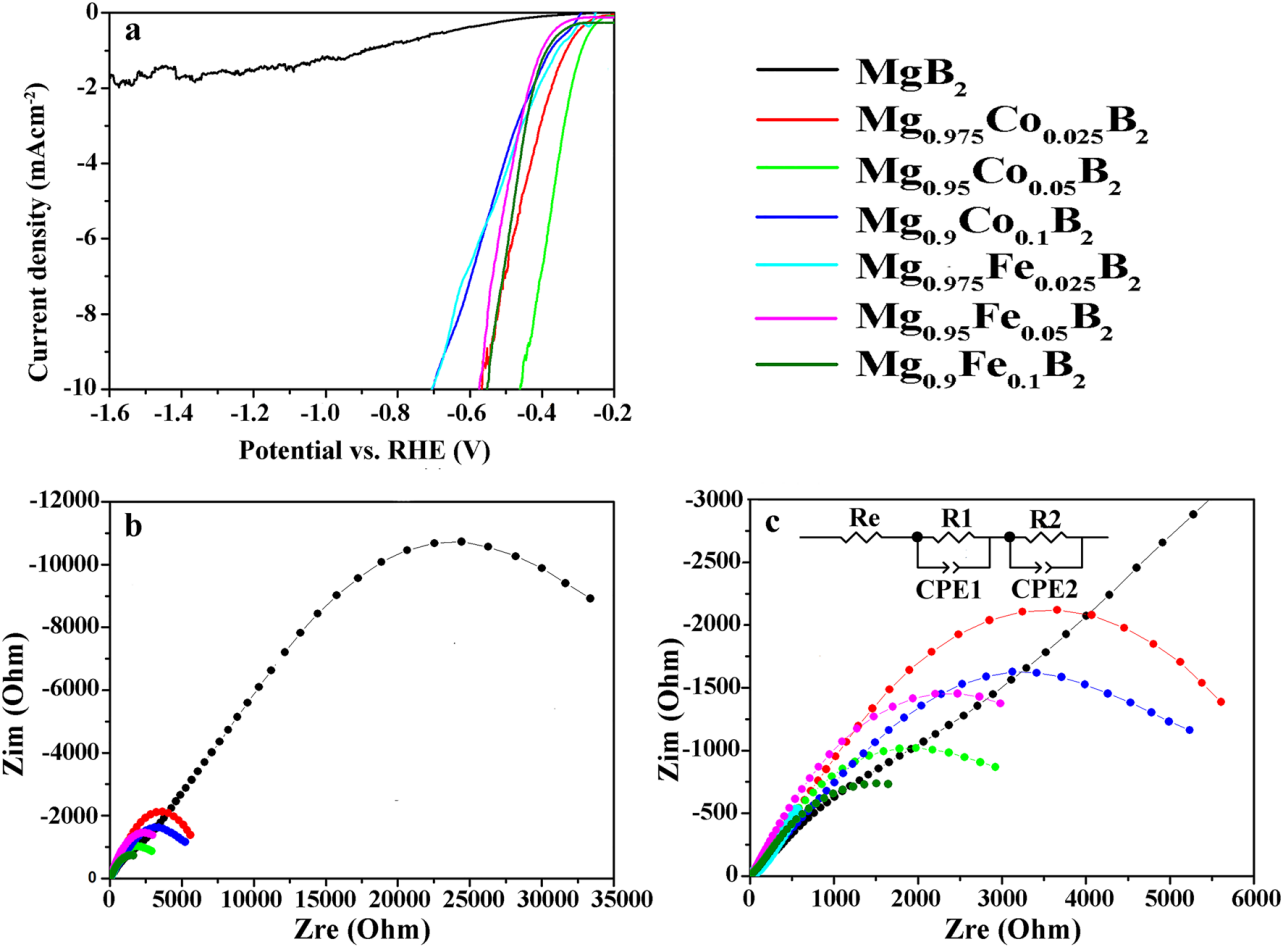
Polarization curves of (**a**) the HER reaction of pure and doped MgB_2_, (**b**, **c**) Nyquist plots measured at OCP in supporting electrolyte of 1 M KOH.

The voltammograms of the HER polarization curves are depicted in Fig. 7a. For this, LSV electrochemical tests were recorded over a range of potential from − 0.2 to − 1.6 V under the basic 1 M KOH medium. It is striking that doping of MgB_2_ with divalent metals affects the HER reaction significantly, as in doped electrodes, the overpotential is extremely lower at − 10 mA cm^−2^ current density compared to that of pure MgB_2_ (see Fig. 7a). In this regard, Mg_0.95_Co_0.05_B_2_ showed the best results in terms of the HER performance with the lowest overpotential at − 10 mA cm^−2^ current density. This suggests that the HER reaction over Mg_0.95_Co_0.05_B_2_ proceeds in an efficient manner which is in accordance with the literature—in that—Co-based borides are favorable catalysts in the water reduction reactions^21^.

As reported by Rafieazad et al.^40^, the presence of MgO impurity can impede the connection between the grains and thereby, undermine the superconductivity of MgB_2_. Moreover, it has been emphasized that coverage of MgB_2_ grains by the MgO layer can diminish the critical current density considerably^40^. With these in mind, Mg_0.95_Co_0.05_B_2_ and Mg_0.9_Fe_0.1_B_2_ displayed the highest HER performance respectively on account of the least amount of impurity in terms of MgO (refer to Fig. 1).

As it was mentioned previously, TMBs possess remarkable features such as reverse electron transfer from boron to metals resulting in fast electrochemical reactions, surface oxidation of TMBs on the account of the formation of active chemicals such borates, boron oxides, TM oxides, TM (oxy)hydroxides leading to effective catalytic reactions, and heat-treatment of TMBs that can promote catalytic performance as a result of phase change^5^. In addition to these aspects, the effect of hetero-metal doping on TMBs has also been widely reviewed. In this context, it was found that metal doping can expand the specific surface area (SSA) through inhibition of the agglomeration that reflects in the exposure of more available active sites, engineering the crystal structures, and tuning the electronic structures^5,33,56^.

It was established that in boride-rich compounds such as FeB_2_, Fe as cation holds a partial positive charge, and B as anion carries a partial negative charge. Here, electron transfer from Fe to B expedites the adsorption of OH^−^ on positively charged Fe sites and H^+^ on negatively charged B sites^55^. Regarding MgB_2_ as parent electrocatalyst utilized in this study, Fe and Co dopants—more electronegative compared to Mg—have changed surface morphology of the samples, regulated electronic structures, and induced fairly higher electron density transferred from cation site to anion, facilitating both HER and OER reactions.

For better interpretation, EIS analyses were performed in open circuit potential to predict charge transfer resistance through the electrodes. Figure 7b,c present the Nyquist plots of all the electrodes in full and zoom screen, respectively. As revealed by FESEM images from the electrode surface (Fig. 5), the fabricated electrodes possess high porosity. To this end, the impedances were modeled using a so-called two-CPE model, R (R CPE) (R CPE), composed of a solution resistance and a connection between two paralleled R-CPE elements in series—the high-frequency R-CPE element is correlated with the porosity whereas the low-frequency element is related to the kinetics of the HER.

In principle, the formation of two semicircles on the Nyquist plot corresponds to the geometric kinetic origin. The first semicircle is related to geometric and the second one is related to kinetic. The diameter of the semicircles represents the charge transfer resistance. Both the transfer of electronic charge on the electrode/electrolyte interface and the charge transfer in the host lattice control the electrochemical reactions at the electrodes. The acquired results for the Nyquist equation by Z-view software are summarized in Table 2 (detailed data in Table S3). The results support that metal doping of MgB_2_ has dramatically decreased both R1 (charge-transfer resistance of the electrode/electrolyte interface) and R2 (charge-transfer resistance of the host lattice), and therefore increased the electrochemical catalytic activity.

**Table 2:** Equivalent-circuit element values for EIS data in the 1 M KOH electrolyte.

Sample	Re (Ω cm^−2^)	R1 (kΩ cm^−2^)	R2 (kΩ cm^−2^)
MgB_2_	130.5	10.993	127.114
Mg_0.975_Co_0.025_B_2_	12.2	1.436	18.331
Mg_0.95_Co_0.05_B_2_	13.6	0.409	10.409
Mg_0.9_Co_0.1_B_2_	13.2	3.539	13.712
Mg_0.975_Fe_0.025_B_2_	7.52	0.454	710
Mg_0.95_Fe_0.05_B_2_	13.5	0.277	14.325
Mg_0.9_Fe_0.1_B_2_	11.9	0.332	7.980

As Mg is a highly reactive metal, it can readily get oxidized and converted into Mg(OH)_2_ on the electrode surface in the KOH solution. The very plain interpretation would be, the formation of Mg(OH)_2_ on the surface can block available active sites, increase the reaction resistance and restrain the diffusion of H atoms into the inner phase. Taken this interpretation into account, the high amount of R1 (10.993 kΩ cm^−2^) in undoped MgB_2_ explains itself. On the contrary, the substitution of Co^2+^ and Fe^2+^ for Mg^2+^ in the alloy reduces the formation of Mg(OH)_2_ and improves the life cycle of the electrode. Additionally, doping amplifies the electron mobility and conductivity of the electrodes, yielding much lower amounts of R2 for the doped electrocatalysts (see Table 2). Overall, Mg_0.9_Fe_0.1_B_2_ (7.980 kΩ cm^−2^) and Mg_0.95_Co_0.05_B_2_ (10.409 kΩ cm^−2^) have the lowest charge-transfer resistance in terms of R2 among the doped electrodes; as a result, they outperform all others in the sense of electrocatalytic performance.

To assess the probable degradation of the electrocatalysts after electrochemical reactions, FESEM coupled with EDS analyses were conducted on the samples. Figure S5 presents the EDS results of the samples after electrochemical reactions at different spots and the outcomes have been reported with and without oxygen content. Based on semi-quantitative analysis results in Tables S1 and S4, it can be observed that there are slight changes in the atomic percent of all elements after HER and OER, implying that the fabricated electrodes have remained largely intact. Considering that reactions have taken place in 1 M KOH, increasing the atomic percent of oxygen on the surface of both best-performing electrodes from 3.63 to 10.59% and 3.12 to 14.93% corresponding to Mg_0.95_Co_0.05_B_2_ and Mg_0.9_Fe_0.1_B_2_ is not surprising. It is assumed that the surface metal hydroxide/oxyhydroxide produced in the course of OER is the major ground for the high water oxidation performance^55^. As a result, high oxygen contents for the post-OER electrodes can be ascribed to Mg-, Fe-, and Co- hydroxide/oxyhydroxide. In addition to this, higher alterations in the atomic percent of metals—Mg, Fe, and Co—can be witnessed compared to nonmetal B. For the case of Mg, it should be noted that inevitably unreacted Mg before HER and OER reactions have been consumed as Mg- hydroxide/oxyhydroxide leading to less Mg contents after electrochemical reactions for both electrodes.

**Figure 8:**
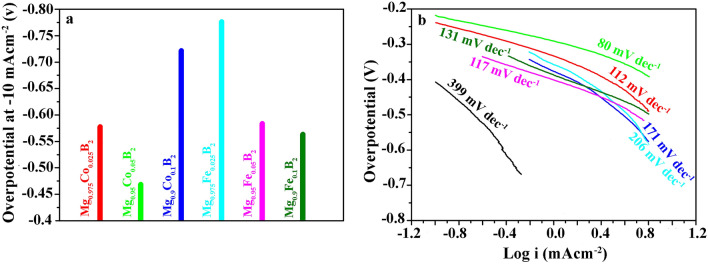
Fundamental electrochemical properties for HER. (**a**) Overpotential at − 10 mA cm^**−**2^ of metal-doped MgB_2_, (**b**) the corresponding Tafel plots of undoped and doped MgB_2_. The colors indicating the same samples pictured in Fig. 7.

For the practical applications in the electrocatalytic process, overpotential should be kept to the lowest amounts to deliver a benchmark current density of 10 mA cm^−2^ during the process. For this reason, metal doping is not only an efficient way to improve the sluggish kinetics of HER but also is beneficial to lower the kinetic energy resistances of electrocatalytic reactions. The overpotentials of Co- and Fe-doped MgB_2_ at − 10 mA cm^−2^ are shown in Fig. 8a indicating the HER performance of the electrodes. Mg_0.95_Co_0.05_B_2_ has a low overpotential of 470 mV at − 10 mA cm^−2^ amidst all the investigated samples owing to its high purity. However, the other Co-doped electrodes did not follow any specific activity trend. In contrast, the activity trend in Fe-doped electrodes showed an inverse relation with Fe content. By increasing Fe amount, overpotential diminished, giving rise to the following trend Mg_0.9_Fe_0.1_B_2_ < Mg_0.95_Fe_0.05_B_2_ < Mg_0.975_Fe_0.025_B_2_.

Concerning the fact that there has been a limited number of reports on layered MDbs in HER, a brief comparison of overpotentials with the present work and recently reported boride based electrocatalysts would be intriguing. For this, Mazánek et al.^1^. stated HER performance of several layered MDbs including MgB_2_ in 0.5 M H_2_SO_4_. According to this report, the best performing MDb was ZrB_2_ with an overpotential of over 950 mV at a current density of − 10 mA cm^−2^. Moreover, an elaborate review was recently published by Gupta et al.^17^. Here, a few results on overpotential of metal borides in 1 M KOH solution are given: Mo–B ≈ 240 mV (20 mA cm^−2^), NiB_*x*_ film ≈ 135 mV (10 mA cm^−2^), Ni–B ≈ 125 mV (20 mA cm^−2^), Co_2_B–CoSe_2_ ≈ 300 mV (10 mA cm^−2^), Co–Ni–B ≈ 205 mV (10 mA cm^−2^), and FeB_2_ ≈ 61 mV (10 mA cm^−2^). Even though at first glance the investigated electrocatalysts in this study appear less competent compared to the above-written non-precious electrocatalysts, it should be overemphasized that the present results emerged with trace amounts of dopants and fairly economical precursors, showing a great promise for future works.

Figure 8b presents Tafel curves for all the synthesized electrodes. The Tafel slope is a crucial indication of reaction kinetics and rate in the HER process^57^. In other words, it is an intrinsic factor for the assessment of the rate-determining steps in the course of HER^58^. According to the previous studies^18,59,60^, two general paths are proposed for hydrogen evolution in alkaline solutions; either based on Volmer–Heyrovsky mechanism (a and b) or the Volmer-Tafel mechanism (c and d):

- H_2_O(1) + e^−^  + * → H* + OH^−^ (aq), Volmer reaction
- H* + H_2_O(1) + e^−^  → H_2_(g) + OH^−^ (aq) + *, Heyrovsky reaction
- H_2_O(1) + e^−^  + * → H* + OH^−^ (aq), Volmer reaction
- H* + H* → H_2_(g) + 2*, Tafel reaction

In the foregoing reactions, * represents available active sites and H* hydrogen atoms bound to active sites. Moreover, it has been estimated that the required Tafel slopes for Volmer reaction, Heyrovsky reaction, and Tafel reaction are ∼120 mV dec^−1^, ∼40 mV dec^−1^, and ∼30 mV dec^−1^, respectively^18^, Regarding the Tafel slopes shown in Fig. 8b, Mg_0.95_Co_0.05_B_2_ has the smallest Tafel slope, 80 mV dec^−1^ which indicates that it falls within the range of 40–120 mV dec^−1^, demonstrating the Volmer-Heyrovsky mechanism. This superior performance accords with overpotential results as well, suggesting facile kinetics and faster electron transport^18,58,61,62^ on Mg_0.95_Co_0.05_B_2_ as compared to all other samples. Interestingly, Mg_0.975_Co_0.025_B_2_ with a Tafel slope of 112 mV dec^−1^, Mg_0.95_Fe_0.05_B_2_ with that of 117 mV dec^−1^, and Mg_0.9_Fe_0.1_B_2_ with a slope of 131 mV dec^−1^ have relatively comparable catalytic activity, proceeding via Volmer mechanism. Other doped samples that displayed rather higher Tafel slopes likely suffered from insufficient conductivity and low accessible active sites^58,61^.

Despite the fact that too high the Tafel slope necessitates the electrocatalyst to provide excessive overpotential to meet the required current density^60^, we cannot draw a clear conclusion based on the relation between overpotential and Tafel slope. Nonetheless, a meaningful point is revealed—that is—at low overpotential (Mg_0.95_Co_0.05_B_2_, Fig. 8a) the Volmer–Heyrovsky reaction is dominated, while at high overpotential (Fe-doped samples, Fig. 8a) the Volmer–Tafel reaction prevails. The preceding remark is substantiated by the literature as well^59^.

The stability tests of the Mg_0.95_Co_0.05_B_2_ and Mg_0.9_Fe_0.1_B_2_ electrodes were performed by the cyclic voltammetry (CV) scanning method. Specifically, the current density was measured over a scanning rate of 50 mV s^−1^ in a voltage window of − 1 V to − 0.2 V (E vs. RHE) for HER under 1 M KOH solution. (see Figure S6). Altogether, for HER stability tests, even though the current density did not decrease appreciably, yet further investigations are needed to excel in the stability of these very new electrocatalysts.

## Experimental

### Synthesis of electrocatalysts

#### Preparation of MgB_2_

In general, there are two methods to synthesize magnesium diboride, namely “in situ” and “ex-situ” techniques. In this study, the former method has been preferred to the latter due to the conspicuous benefits. In the “in situ’’ technique, precursor materials are mixed with stoichiometric compositions and annealed under the inert condition. Compared to the “ex-situ” technique. “in situ” is more effective for doping purposes, since already reacted powders of MgB_2_ are used in the “ex-situ” technique which does not leave enough room to develop metal doping^40^. Further, according to the literature^40^, “ex-situ” fabrication of MgB_2_ is usually subjected to poor grain conductivity which hinders its application in electrolysis. In brief, the synthesis of pure MgB_2_ powders was carried out via the “in situ” process. A specified amount of magnesium powder (Mg, 99.8%; Alfa Aesar) and amorphous nano boron (B, 98.5%; Pavezyum) were mixed in stoichiometric amounts in a glove box under Ar atmosphere and transferred into a steel tube. Afterward, the steel tube was sealed by arc melting under Ar. Then, the annealing process was applied at 850 °C for 6 h. It should be mentioned that following the annealing process, powders were ground in an agate mortar to obtain fine and homogenous powders for the electrochemical measurements.

#### Preparation of Mg_1-*x*_*TM*_*x*_B_2_ (*x* = 0.025, 0.05, and 0.1; *TM* = Fe and Co)

**Figure 9:**
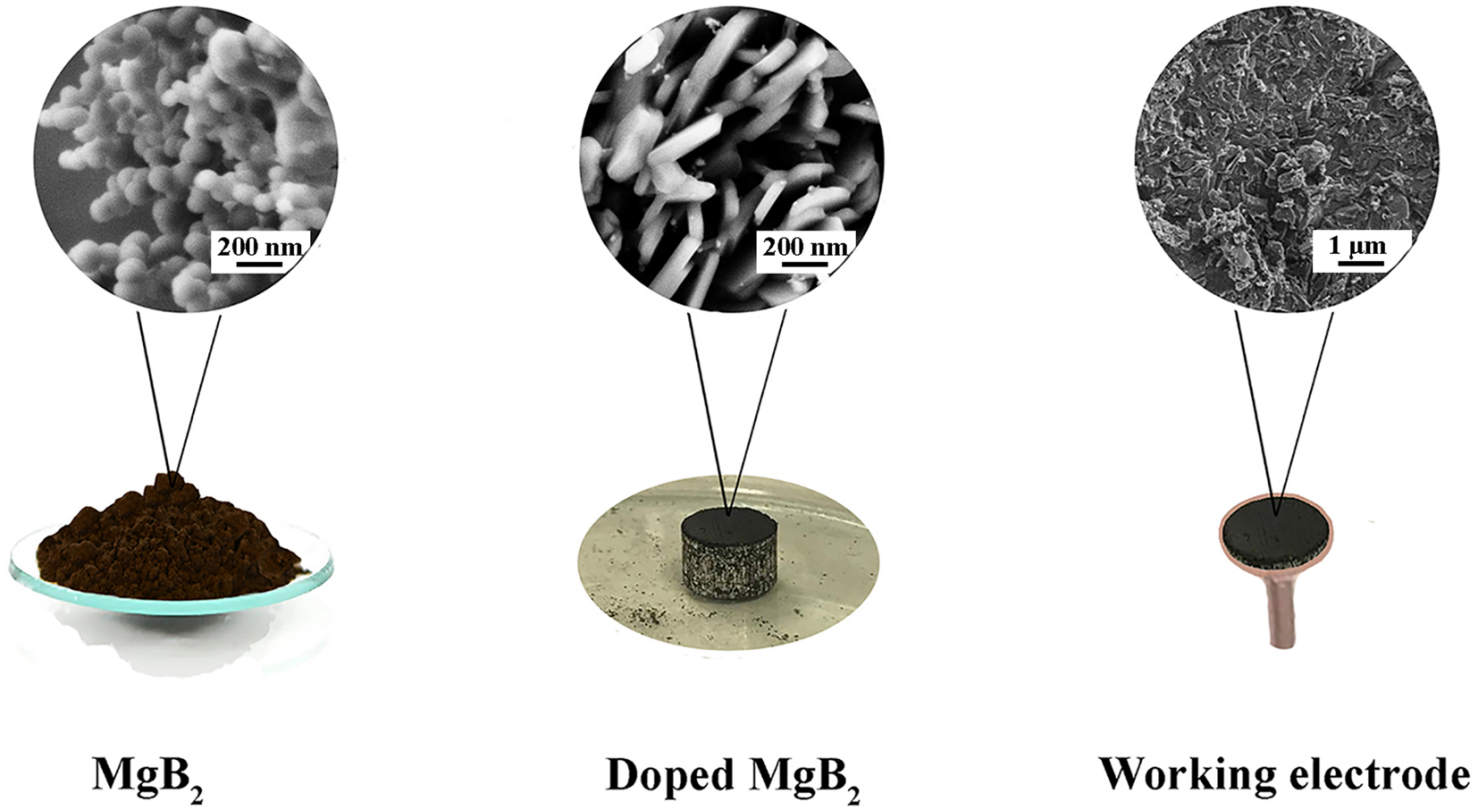
Illustration of final states of MgB_2_ powder, doped MgB_2_ pellets, and the working electrode.

For the substitution experiments, cobalt powder (Co, 99.8%; Alfa Aesar), and reduced fine powder of iron (Fe, ≥ 99%, Sigma-Aldrich), were utilized individually. The powders; Mg, Fe (or Co), and B with the formal composition of Mg_1-*x*_*TM*_*x*_B_2_ were homogenized by grinding in an agate mortar and then compacted into pellets with a diameter of 10 mm at a pressure of about 10 ton. The pellets were then sealed inside a steel tube and went under heat-treatment at 850 °C for 6 h. The post-annealing process is the same as bare MgB_2_^63,64^ (refer to Fig. 9).

### Apparatus and procedure

#### Apparatus

The crystal structure and the phase purity analysis of the samples were carried out by X-ray diffraction (XRD, Rigaku Mini Flex 600) with Cu Kα (λ = 1.5418 Å) radiation (40 kV voltage and 15 mA). Diffraction patterns were collected between 20° and 85° of 2θ. A zero-background powder specimen holder was utilized for the XRD analysis of all samples. The lattice parameters were refined using the WinCSD program package^65^. High-resolution X-ray photoelectron spectroscopy (XPS) was performed with a Thermo K-Alpha with an Al Kα source. The fittings were accomplished with Avantage software. All peaks were corrected with respect to the C1s peak at 284.5 eV. For the analysis of the microstructure and surface morphology coupled with chemical composition, Field Emission Scanning Electron Microscopy (FESEM) equipped with Energy Dispersive X-ray Spectroscopy (EDS) analyses were employed by Zeiss Ultra Plus Field Emission Scanning Electron Microscope using accelerating voltages of 5 and 12 kV, separately.

#### Electrochemical measurements

Electrochemical measurements of the prepared electrocatalysts were conducted in a 1 M KOH solution using a three-electrode cell equipped with standard Reduced Hydrogen Electrode (RHE; HydroFLEX) as reference electrode and Pt wire as a counter electrode. All experiments were evaluated at room temperature using a VersaSTAT Potentiostat Galvanostat. The working electrodes were made on a copper foil with a surface area of 0.35 cm^2^ via deposition of powders through a polymeric binder. In brief, 30 mg of powders were mixed with a drop of the polymeric binder (paraffin wax) at about 100 °C and anchored on the copper foil supported by copper wire with a diameter of 1 mm. Then, the mixture was dried at ambient temperature. Finally, the designed electrodes were covered by a tubular plastic to prevent any contact with the solution. It is worth noting that the powder was dispersed uniformly on the foil and covered the whole area completely. For the electrochemical measurements, 0.5 g (powder) per mL (solution) was used for each electrode (see Fig. 9).

For electrochemical measurements, each sample was undergone Open Circuit Potential (OCP) measurements for 900 s to stabilize in the electrolyte. Then Electrochemical Impedance Spectroscopy (EIS) was used to evaluate the charge transfer process as the critical factor determining the electrocatalytic activity on the electrodes. The OCP potential was recorded from a frequency of 100 kHz to 0.1 Hz with a 10 mV rms sinusoidal modulation. Afterward, the HER electrocatalytic activity was measured by applying Linear Sweep Voltammetry (LSV) method from − 0.2 to − 1.6 V at a sweep rate of 5 mV s^−1^ towards HER catalysis. Also, overpotential calculation was performed at − 10 mA cm^−2^ as an index of HER performance. For the OER, LSV was recorded at the same rate as HER from 0 to + 1.8 V towards OER catalysis.

## Conclusions

In this work, we performed a straightforward scalable method to synthesize a series of novel metal-doped MgB_2_ comprising earth-abundant elements, Fe and Co, with a loading concentration of 0.025, 0.05, and 0.1. The as-prepared materials anchored on a copper foil were employed as electrodes for the water-splitting process to function as HER electrocatalysts. Meanwhile, they were examined as OER catalysts as well. The overall outcomes substantiated that introduction of Co and Fe into the structure of layered MgB_2_ meaningfully enhanced both OER and HER performance. In this way, a remarkable improvement of OER activity for Fe-doped MgB_2_ electrodes was ascribed to the dramatic reduction of charge-transfer resistance both at electrode/electrolyte surface and within the host lattice. As a result, increasing Fe content gave rise to outstanding OER activity corresponding to Mg_0.9_Fe_0.1_B_2_, by virtue of its optimum values of charge-transfer resistance. Besides, the best-performing catalyst in terms of HER was obtained for Mg_0.95_Co_0.05_B_2_ displaying an overpotential of 470 mV at − 10 mA cm^−2^ and a small Tafel slope of 80 mV dec^−1^. This notable performance arose from several factors including the efficacious integration of Co in the crystal structure, facile kinetics, and fast electron transport that resulted in a drastic abatement of charge-transfer resistance in the host lattice compared to the undoped sample (10.409 vs. 127.114 kΩ cm^−2^). This comprehensive electrochemical study conducted on Fe- and Co-doped MgB_2_ may provide further insights into designing new earth-abundant and efficient materials that rival and exceed the performance of conventional electrocatalysts.

## Supplementary Information

Supplementary Information.

## References

[1] Mazánek V, Nahdi H, Luxa J, Sofer Z, Pumera M (2018). Electrochemistry of layered metal diborides. Nanoscale.

[2] Apak S, Atay E, Tuncer G (2017). Renewable hydrogen energy and energy efficiency in Turkey in the 21st century. Int. J. Hydrog. Energy.

[3] Xiong B, Chen L, Shi J (2018). Anion-containing noble-metal-free bifunctional electrocatalysts for overall water splitting. ACS Catal..

[4] Ghadge SD (2019). Experimental and theoretical validation of high efficiency and robust electrocatalytic response of one-dimensional (1D) (Mn, Ir)O 2:10F nanorods for the oxygen evolution reaction in pem-based water electrolysis. ACS Catal..

[5] Chen Z (2020). Boride-based electrocatalysts: Emerging candidates for water splitting. Nano Res..

[6] Sun H (2018). Superhydrophilic amorphous Co-B-P nanosheet electrocatalysts with Pt-like activity and durability for the hydrogen evolution reaction. J. Mater. Chem. A.

[7] Yang H, Ma Y, Lv X, Huang B, Dai Y (2020). Prediction of intrinsic electrocatalytic activity for hydrogen evolution reaction in Ti4X3 (X = C, N). J. Catal..

[8] Nsanzimana JMV (2019). facile synthesis of amorphous ternary metal borides-reduced graphene oxide hybrid with superior oxygen evolution activity. ACS Appl. Mater. Interfaces.

[9] Feng T (2019). Morphological and interfacial engineering of cobalt-based electrocatalysts by carbon dots for enhanced water splitting. ACS Sustain. Chem. Eng..

[10] Wang AL, Xu H, Li GR (2016). NiCoFe layered triple hydroxides with porous structures as high-performance electrocatalysts for overall water splitting. ACS Energy Lett..

[11] Kim YK, Kim JH, Jo YH, Lee JS (2019). Precipitating metal nitrate deposition of amorphous metal oxyhydroxide electrodes containing Ni, Fe, and Co for electrocatalytic water oxidation. ACS Catal..

[12] Yu J (2019). Recent Advances and Prospective in Ruthenium-Based Materials for Electrochemical Water Splitting. ACS Catal..

[13] Ji L, Wang J, Teng X, Meyer TJ, Chen Z (2020). CoP nanoframes as bifunctional electrocatalysts for efficient overall water splitting. ACS Catal..

[14] Liang K (2017). Overall water splitting with room-temperature synthesized NiFe oxyfluoride nanoporous films. ACS Catal..

[15] Wang X (2018). Amorphous multi-elements electrocatalysts with tunable bifunctionality toward overall water splitting. ACS Catal..

[16] Sun H (2019). Morphological and electronic tuning of Ni2P through iron doping toward highly efficient water splitting. ACS Catal..

[17] Gupta S, Patel MK, Miotello A, Patel N (2020). Metal boride-based catalysts for electrochemical water-splitting: A review. Adv. Funct. Mater..

[18] Kuang P, Tong T, Fan K, Yu J (2017). In situ fabrication of Ni-Mo bimetal sulfide hybrid as an efficient electrocatalyst for hydrogen evolution over a wide pH range. ACS Catal..

[19] Wang D, Song Y, Zhang H, Yan X, Guo J (2020). Recent advances in transition metal borides for electrocatalytic oxygen evolution reaction. J. Electroanal. Chem..

[20] Zhu Y (2017). Enhancing electrocatalytic activity for hydrogen evolution by strongly coupled molybdenum nitride@nitrogen-doped carbon porous nano-octahedrons. ACS Catal..

[21] You B, Sun Y (2018). Innovative strategies for electrocatalytic water splitting. Acc. Chem. Res..

[22] Chaudhari NK, Jin H, Kim B, Lee K (2017). Nanostructured materials on 3D nickel foam as electrocatalysts for water splitting. Nanoscale.

[23] Gu X-K, Carl A, Camayang J, Samira S, Nikolla E (2020). Oxygen evolution electrocatalysis using mixed metal oxides under acidic conditions: challenges and opportunities. J. Catal..

[24] Li Y (2020). Recent advances on water-splitting electrocatalysis mediated by noble-metal-based nanostructured materials. Adv. Energy Mater..

[25] Zhao Q (2018). Tuning electronic push/pull of Ni-based hydroxides to enhance hydrogen and oxygen evolution reactions for water splitting. ACS Catal..

[26] Chung DY (2017). Large-scale synthesis of carbon-shell-coated FeP nanoparticles for robust hydrogen evolution reaction electrocatalyst. J. Am. Chem. Soc..

[27] Wu Y, Gao Y, He H, Zhang P (2019). Novel electrocatalyst of nickel sulfide boron coating for hydrogen evolution reaction in alkaline solution. Appl. Surf. Sci..

[28] Qi P (2020). Active nickel derived from coordination complex with weak inter/intra-molecular interactions for efficient hydrogen evolution via a tandem mechanism. J. Catal..

[29] Zhang P (2016). Electroless plated Ni-Bx films as highly active electrocatalysts for hydrogen production from water over a wide pH range. Nano Energy.

[30] Gupta S, Patel N, Miotello A, Kothari DC (2015). Cobalt-boride : An efficient and robust electrocatalyst for hydrogen evolution reaction. J. Power Sources.

[31] Park H, Encinas A, Scheifers JP, Zhang Y, Fokwa BPT (2017). Hydrogen evolution hot paper boron-dependency of molybdenum boride electrocatalysts for the hydrogen evolution reaction. Angewandte.

[32] Zhang R (2018). Engineering cobalt defects in cobalt oxide for highly efficient electrocatalytic oxygen evolution. ACS Catal..

[33] Guo F (2019). A class of metal diboride electrocatalysts synthesized by a molten salt-assisted reaction for the hydrogen evolution reaction. Chem. Commun..

[34] Chen Z (2018). Study of cobalt boride-derived electrocatalysts for overall water splitting. Int. J. Hydrogen Energy.

[35] Das SK, Jasuja K (2018). Chemical exfoliation of layered magnesium diboride to yield functionalized nanosheets and nanoaccordions for potential flame retardant applications. ACS Appl. Nano Mater..

[36] Chung HY, Weinberger MB, Yang JM, Tolbert SH, Kaner RB (2008). Correlation between hardness and elastic moduli of the ultraincompressible transition metal diborides RuB#!2, OsB2, and ReB2. Appl. Phys. Lett..

[37] Brutti S, Gigli G (2009). Order-disorder transition and phase separation in the MgB2 metallic sublattice induced by Al doping. J. Chem. Theory Comput..

[38] Yamamoto A (2005). Effects of B4C doping on critical current properties of MgB 2 superconductor. Supercond. Sci. Technol..

[39] Cristina B, Tsutomu Y (2001). Review of the superconducting properties of MgB2. Supercond. Sci. Technol..

[40] Rafieazad M, Balcı Ö, Acar S, Somer M (2017). Review on magnesium diboride (MgB2) as excellent superconductor: Effects of the production techniques on the superconducting properties. J. Boron.

[41] Kortus J, Mazin II, Belashchenko KD, Antropov VP, Boyer LL (2001). Superconductivity of metallic Boron in MgB2. Phys. Rev. Lett..

[42] Nagamatsu J, Nakagawa N, Muranaka T, Zenitani Y, Akimitsu J (2001). Superconductivity at 39 K in magnesium diboride. Nature.

[43] Ansari IA (2019). Study of dynamic behaviors for nano Fe-doped MgB2 superconductor via ac-susceptibility measurements. Ceram. Int..

[44] Mazin II, Antropov VP (2002). Electronic structure, electron-phonon coupling, and multiband effects in MgB2. Phys. C Supercond..

[45] Talapatra A (2005). X-ray photoelectron spectroscopy studies of MgB2 for valence state of Mg. Phys. C Supercond. Appl..

[46] Shannon RD (2016). Revised effective ionic radii and systematic study of inter atomic distances in halides and chalcogenides in halides and chaleogenides. Acta Cryst.

[47] Corneille JS, He JW, Goodman DW (1994). XPS characterization of ultra-thin MgO films on a Mo (100) surface. Surf. Sci..

[48] Yao HB, Li Y, Wee ATS (2000). An XPS investigation of the oxidation r corrosion of melt-spun Mg. Appl. Surf. Sci..

[49] Sanz, J. M., Tyuliev, G. T., Morant, C., Soriano, L., Espinos, J. P., Fernández, A. & Gonzalez-Elipe, A. R. Electronic structure of transition metal oxide nanostructures. *J. Surf. Anal.* **3**(2), 279–285 (1997).

[50] Sanz, J. M., Nunez, R., Fuentes, G. G., Soriano, L. & Morant, C. Characterization of nanostructures by electron spectroscopies. *J. Surf. Anal* **5**(2), 338–342 (1994).

[51] Taleatu BA (2014). XPS and some surface characterizations of electrodeposited MgO nanostructure. Surf. Interface Anal..

[52] Seyama H, Soma M (1984). X-ray photoelectron spectroscopic study of montmorillonite containing exchangeable divalent cations. J. Chem. Soc. Faraday Trans. Phys. Chem. Condens. Phases.

[53] Kuzmann E (2002). Local environments of iron and cobalt in doped MgB2 superconductors. Supercond. Sci. Technol..

[54] Sheng M (2018). Network-like porous Co-Ni-B grown on carbon cloth as efficient and stable catalytic electrodes for hydrogen evolution. Electrochem. Commun..

[55] Li H (2017). Earth-abundant iron diboride (FeB2) nanoparticles as highly active bifunctional electrocatalysts for overall water splitting. Adv. Energy Mater..

[56] Nsanzimana JMV (2018). An efficient and earth-abundant oxygen-evolving electrocatalyst based on amorphous metal borides. Adv. Energy Mater..

[57] Yan S (2020). Ultrafine Co:FeS2/CoS2 heterostructure nanowires for highly efficient hydrogen evolution reaction. ACS Appl. Energy Mater..

[58] Han X (2018). Hydrogen evolution reaction on hybrid catalysts of vertical MoS2 nanosheets and hydrogenated graphene. ACS Catal..

[59] Zheng Y (2014). Hydrogen evolution by a metal-free electrocatalyst. Nat. Commun..

[60] Laursen AB, Kegnæs S, Dahl S, Chorkendorff I (2012). Molybdenum sulfides—Efficient and viable materials for electro—And photoelectrocatalytic hydrogen evolution. Energy Environ. Sci..

[61] Li Q, Xing Z, Wang D, Sun X, Yang X (2016). In situ electrochemically activated CoMn-S@NiO/CC nanosheets array for enhanced hydrogen evolution. ACS Catal..

[62] Hu Y (2019). Efficient hydrogen evolution activity and overall water splitting of metallic Co4N nanowires through tunable d-orbitals with ultrafast incorporation of FeOOH. ACS Appl. Mater. Interfaces.

[63] Kühberger M, Gritzner G (2002). Effects of Sn, Co and Fe on MgB2. Phys. C Supercond. Appl..

[64] Moritomo, Y. & Xu, S. Effects of transition metal doping in MgB$_2$ superconductor. **2,** (2001).

[65] Akselrud L, Grin Y (2014). WinCSD: Software package for crystallographic calculations (Version 4). J. Appl. Crystallogr..

