# Supplementary material for: Metal doped layered MgB2 nanoparticles as novel electrocatalysts for water splitting
Source: Sci Rep. 2021 Feb 8;11:3337. doi: 10.1038/s41598-021-83066-7 (PMC7870881; doi:10.1038/s41598-021-83066-7)
Supplement: Supplementary file 1 — Supplementary Information. [file 41598_2021_83066_MOESM1_ESM.docx]

**Supporting Information**

**Metal Doped Layered MgB_2_ Nanoparticles as Novel Electrocatalysts for Water Splitting**

Ebrahim Sadeghi^a,b^, Naeimeh Sadat Peighambardoust^a^, Masoumeh Khatamian^c^, Ugur Unal^d,e^, ‎and Umut Aydemir*^a,e^

^a^ Koç University Boron and Advanced Materials Application and Research Center (KUBAM), Sariyer, Istanbul, 34450, Turkey,

^b^ Graduate School of Sciences and Engineering, Koç University, Sariyer, Istanbul, 34450, Turkey.

^c^ Inorganic Chemistry Department, Faculty of Chemistry, University of Tabriz, 5166616471, Iran

^d^ Koç University Surface Science and Technology Center (KUYTAM), Sariyer, Istanbul, 34450, Turkey

^e^ Department of Chemistry, Koç University, Sariyer, Istanbul, 34450, Turkey

^*^ Corresponding author:

Email: [uaydemir@ku.edu.tr](mailto:uaydemir@ku.edu.tr)

***SEM EDS Analysis Data Before HER/OER***


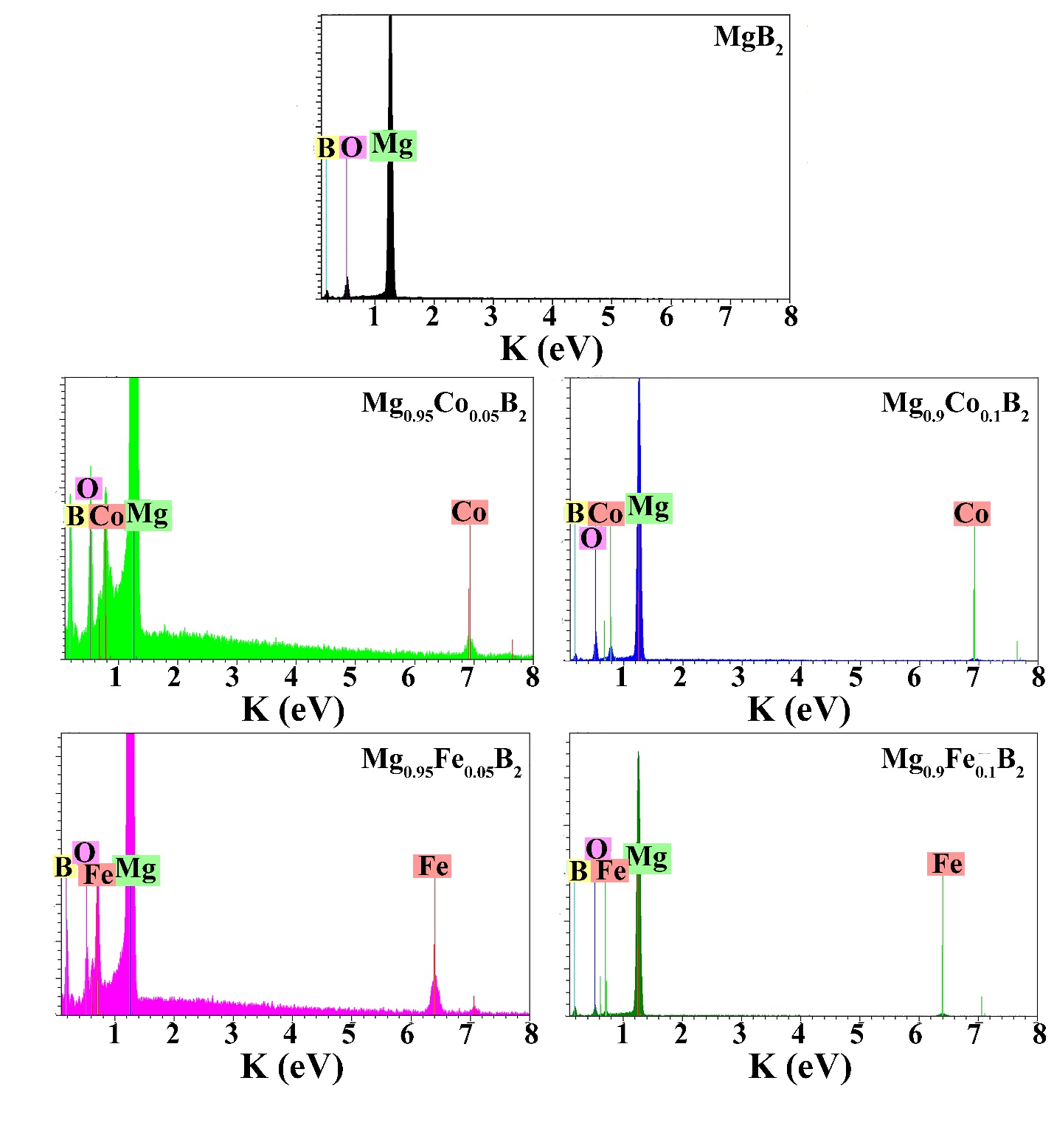


**Figure S1.** EDS analysis graph of pure MgB_2_ and Mg_1-_*_x_Tm_x_*B_2_ (*x* = 0.05, and 0.1; *Tm* = Fe and Co) before electrochemical reactions.

**Table S1.** Energy-dispersive X-ray spectroscopy (EDS) analysis of samples before HER and OER.

| Sample | Element | atomic % | atomic % |
| --- | --- | --- | --- |
| MgB_2_ | Mg  B  O | 30.51  69.49  - | 29.28  63.26  7.46 |
| Mg_0.95_Fe_0.05_B_2_ | Mg  Fe  B  O | 26.65  1.32  72.03  - | 26.12  1.29  69.58  3.01 |
| Mg_0.95_Co_0.05_B_2_ | Mg  Co  B  O | 26.22  1.59  72.19  - | 25.62  1.55  69.20  3.63 |
| Mg_0.9_Fe_0.1_B_2_ | Mg  Fe  B  O | 27.20  2.31  70.49  - | 26.63  2.26  67.99  3.12 |
| Mg_0.9_Co_0.1_B_2_ | Mg  Co  B  O | 31.45  3.23  65.32  - | 29.54  3.00  58.40  9.06 |

**Table S2.** Lattice parameters (*a* and *c* in the space group *P*6/*mmm*) of samples investigated.

| Starting Composition | *a* (Å) | *c* (Å) |
| --- | --- | --- |
| MgB_2_ | 3.0832(3) | 3.5187(5) |
| Mg_0.975_Co_0.025_B_2_ | 3.0847(2) | 3.5223(4) |
| Mg_0.95_Co_0.05_B_2_ | 3.0864(2) | 3.5241(3) |
| Mg_0.9_Co_0.1_B_2_ | 3.0834(3) | 3.5219(6) |
| Mg_0.975_Fe_0.025_B_2_ | 3.0843(3) | 3.5206(7) |
| Mg_0.95_Fe_0.05_B_2_ | 3.0849(1) | 3.5240(3) |
| Mg_0.9_Fe_0.1_B_2_ | 3.0842(2) | 3.5238(4) |

***XPS Spectra***


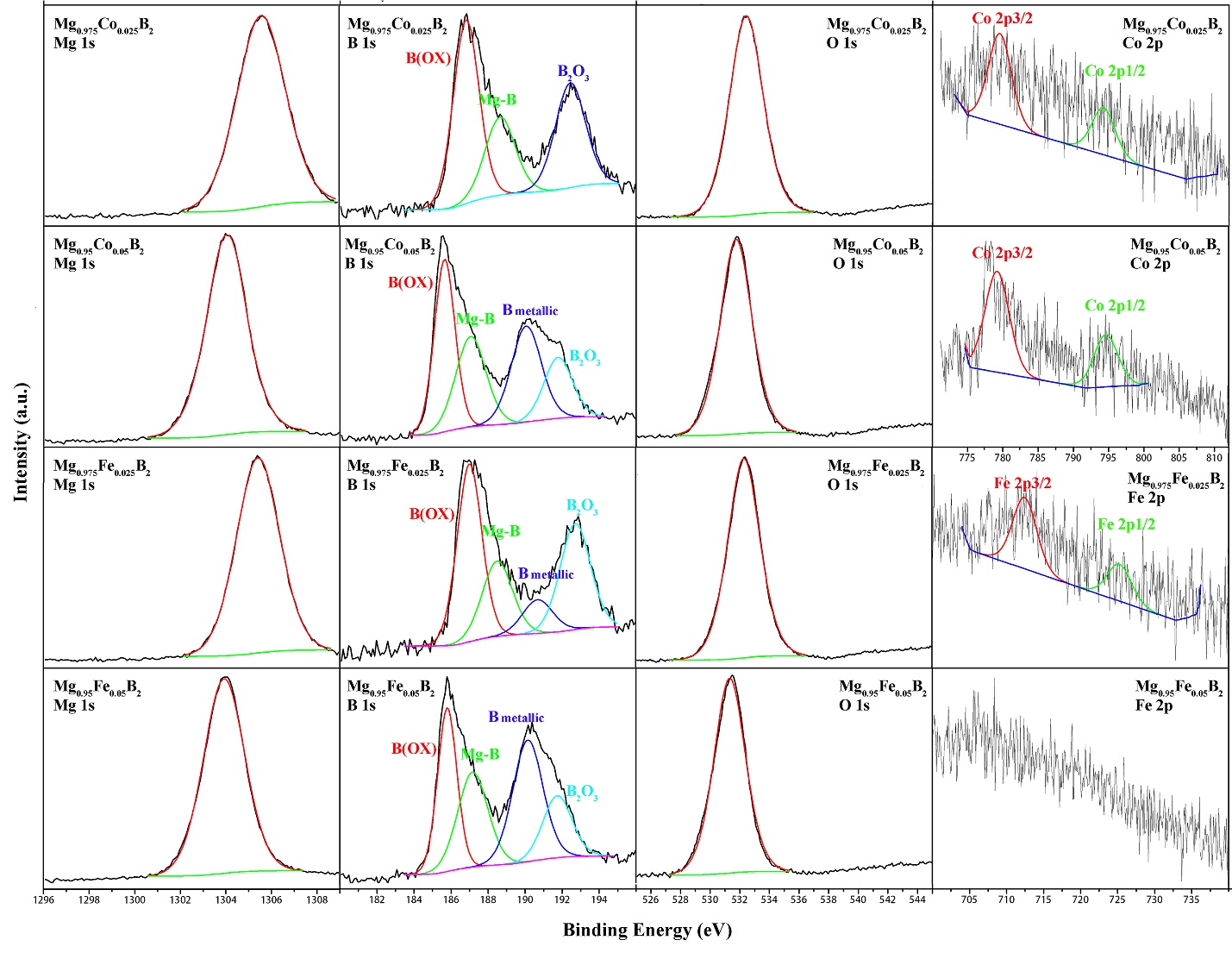


**Figure S2.** XPS spectra of Mg 1s, B 1s, O 1s, Co 2p, and F e 2p in Mg_0.975_Co_0.025_B_2_, Mg_0.95_Co_0.05_B_2_, Mg_0.975_Fe_0.025_B_2_, and Mg_0.95_Fe_0.05_B_2_.

***EDS Mapping***


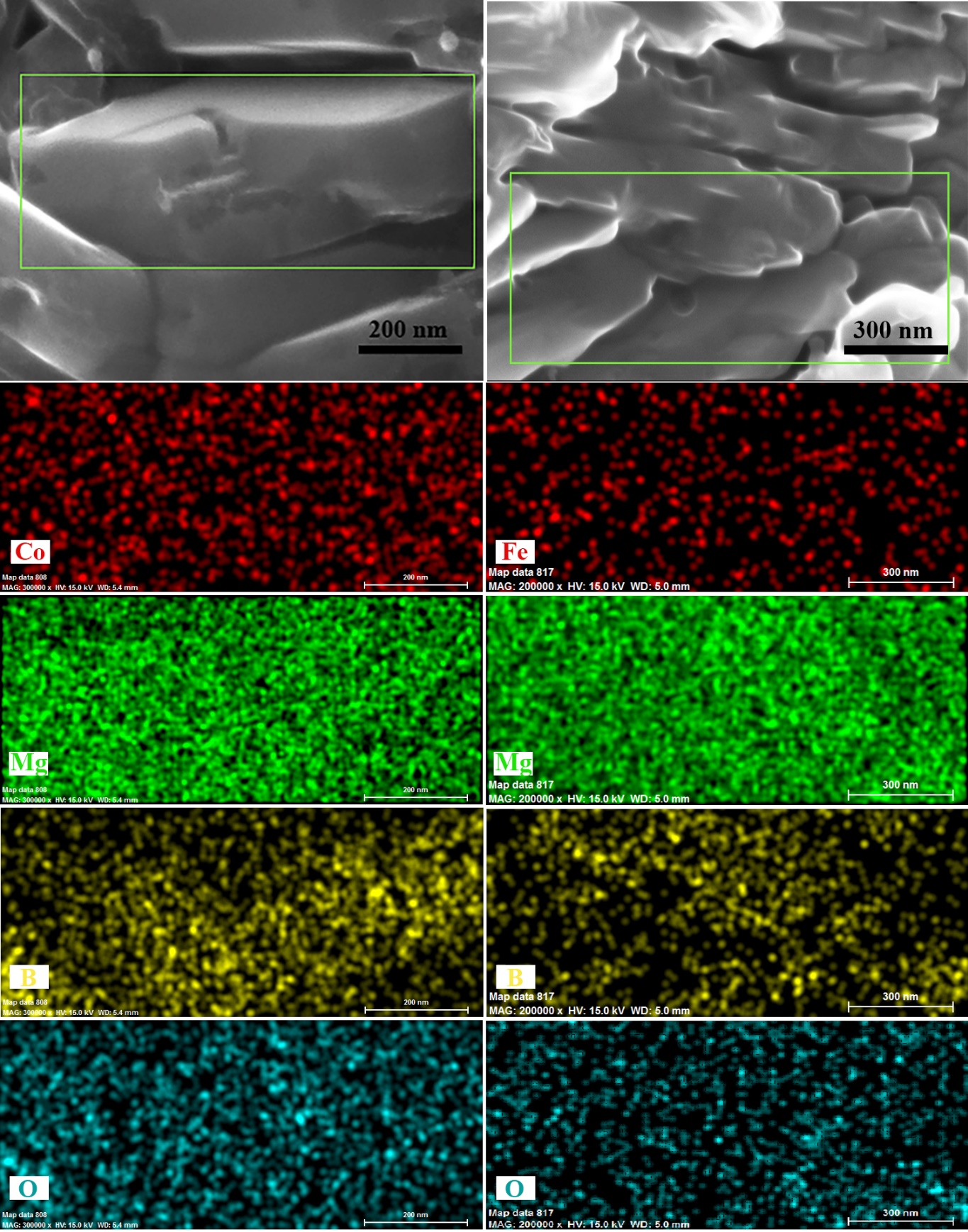


**Figure S3.** SEM images (15.0 kV HV) of Mg_0.95_Co_0.05_B_2_ (left column) and Mg_0.9_Fe_0.1_B_2_ (right column) samples with corresponding elemental mappings.

***SEM Images***


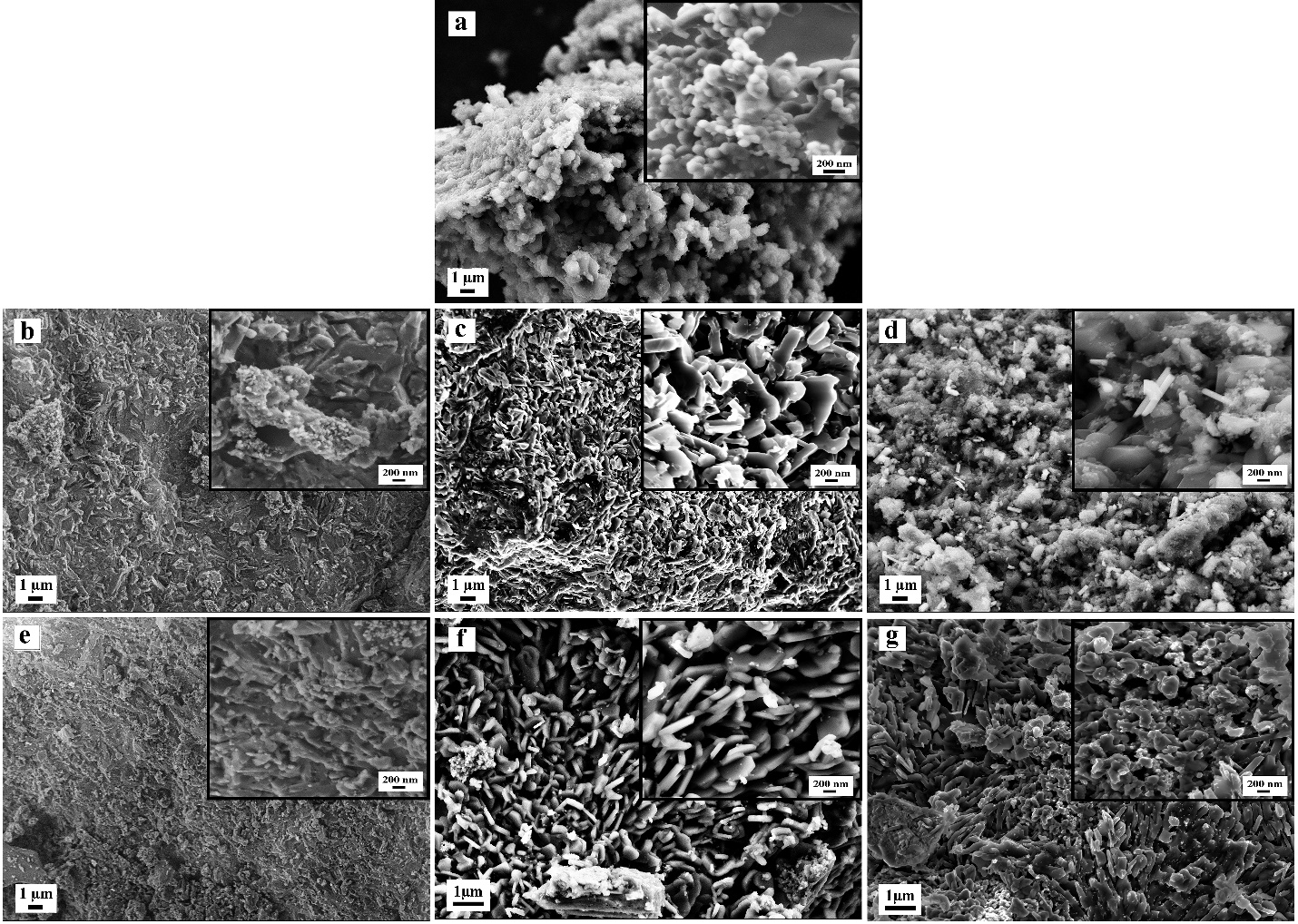


**Figure S4.** SEM images of a) pure MgB_2_, b) Mg_0.975_Co_0.025_B_2_, c) Mg_0.95_Co_0.05_B_2_, d) Mg_0.9_Co_0.1_B_2_, e) Mg_0.975_Fe_0.025_B_2_, f) ‎Mg_0.95_Fe_0.05_B_2_, and g) Mg_0.9_Fe_0.1_B_2_‎

***EIS Data***

**Table S3.** Equivalent-circuit element values for EIS data in the 1 M KOH electrolyte.

|  | **Re (Ωcm^-2^)** | **R1**  **(kΩ cm^-2^)** | **CPE1-T**  **(Fs^p-1^**  **cm^-2^)** | **CPE1-P** | **R2**  **(****kΩ cm^-2^)** | **CPE2-T**  **(Fs^p-1^**  **cm^-2^)** | **CPE2-P** |
| --- | --- | --- | --- | --- | --- | --- | --- |
| MgB_2_ | 130.5 | 10.993 | 2.5E-05 | 0.43 | 127.114 | 5.1E-05 | 0.56 |
| Mg_0.975_Co_0.025_B_2_ | 12.2 | 1.436 | 4.5E-04 | 0.47 | 18.331 | 2.4E-04 | 0.72 |
| Mg_0.95_Co_0.05_B_2_ | 13.6 | 0.409 | 1.3E-03 | 0.45 | 10.409 | 5.1E-04 | 0.66 |
| Mg_0.9_Co_0.1_B_2_ | 13.2 | 3.539 | 3.4E-04 | 0.45 | 13.712 | 2.7E-04 | 0.71 |
| Mg_0.975_Fe_0.025_B_2_ | 7.52 | 0.454 | 2.7E-04 | 0.36 | 710 | 1.5E-05 | 0.56 |
| Mg_0.95_Fe_0.05_B_2_ | 13.5 | 0.277 | 3.4E-03 | 0.40 | 14.325 | 6.2E-04 | 0.67 |
| Mg_0.9_Fe_0.1_B_2_ | 11.9 | 0.332 | 1.6E-03 | 0.45 | 7.980 | 1.2E-03 | 0.62 |

***SEM EDS Analysis Data After HER/OER***


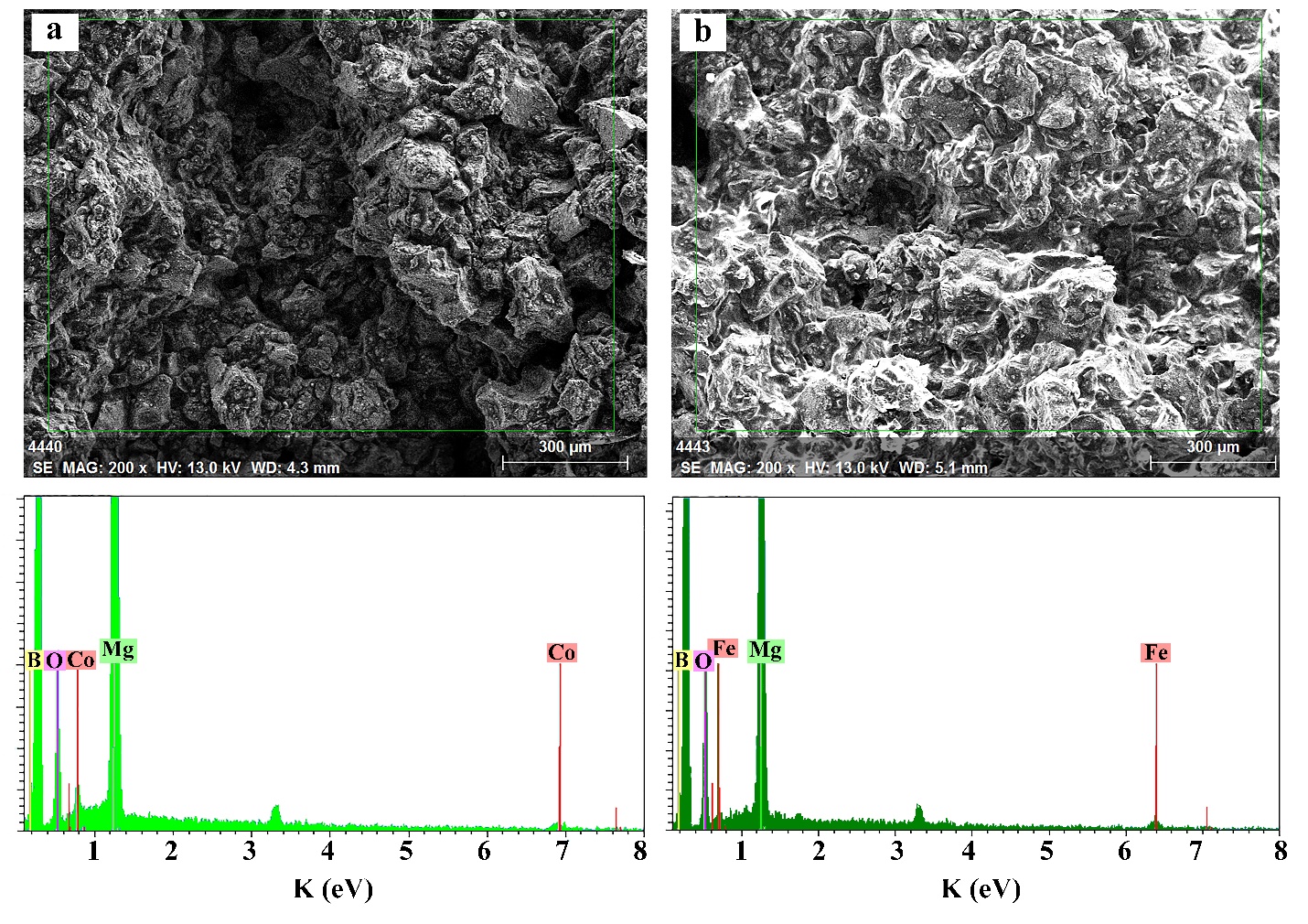


**Figure S5.** EDS results of the (a) Mg_0.95_Co_0.05_B‎_2_ and (b) Mg_0.9_Fe_0.1_B_2_ after electrochemical reactions.

**Table S4.** Energy-dispersive X-ray spectroscopy (EDS) analysis of the best-performing samples after HER and OER.

| Sample | Element | atomic % | atomic % |
| --- | --- | --- | --- |
| Mg_0.95_Co_0.05_B_2_ | Mg  Co  B  O | 25.29  1.19  73.52  - | 23.65  1.09  64.68  10.59 |
| Mg_0.9_Fe_0.1_B_2_ | Mg  Fe  B  O | 21.71  1.64  76.65  - | 19.59  1.48  64.00  14.93 |

***CV Stability Tests***

Aside from catalytic activity, stability is another influential criterion. To appraise the cycling durability of the Mg_0.95_Co_0.05_B_2_ and Mg_0.9_Fe_0.1_B_2_ electrodes, the cyclic voltammetry (CV) plots were measured within a potential range of -1 and -0.2 V for 1000 cycles at a scan rate of 50 mV s^-1^. Fig. S6a and b demonstrate the reordered CV curves of Mg_0.95_Co_0.05_B_2_ and Mg_0.9_Fe_0.1_B_2_ electrodes for the 1^st^ and 1000^th^ cycles in 1 M KOH electrolyte. As shown in the figure, it is obvious that after 1000 cycles the current density diminished for both electrodes. Even though the current density did not decrease appreciably, yet further investigations are needed to excel in the stability of these very new electrocatalysts.


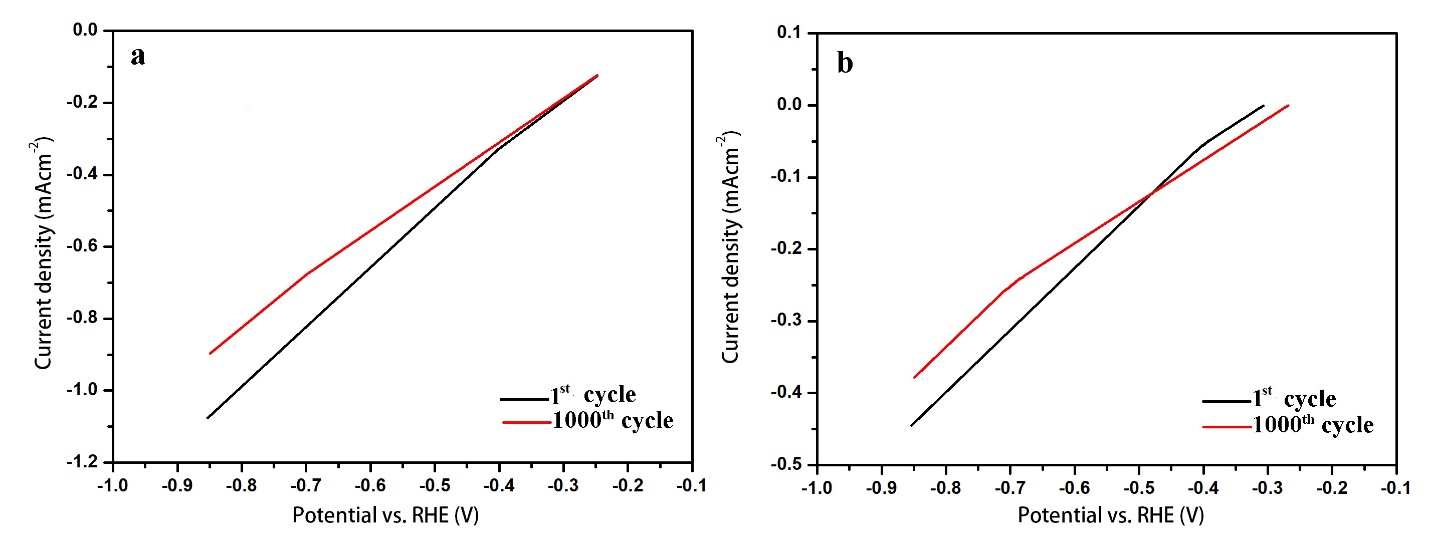


**Figure S6.** Electrochemical cycling stability measurement. a) and b) CV curves of Mg_0.95_Co_0.05_B_2_ and Mg_0.9_Fe_0.1_B_2_, respectively, for 1^st^ and 1000^th^ cycles in a 1 M KOH electrolyte at a scan rate of 50 mV s^−1^.
